# Prevalence and Determinants of Endothelial Dysfunction among Adults Living with HIV in Northwest Nigeria

**DOI:** 10.5334/gh.1264

**Published:** 2023-10-20

**Authors:** Aisha M. Nalado, Bala Waziri, Anas Ismail, Nafiu Umar, Zainab U. Ibrahim, Patience Obiagwu, Baba M. Musa, Mahmoud U. Sani, Aliyu Abdu, Faisal S. Dankishiya, Mansur A. Ramalan, Hadiza Saidu, Usman J. Wudil, C. William Wester, Muktar H. Aliyu

**Affiliations:** 1 Department of Medicine, Bayero University, Kano & Aminu Kano Teaching Hospital, Kano, NG; 2 Department of Medicine, Renal Unit, Ibrahim Badamasi Babangida Specialist Hospital, Minna, NG; 3 Department of Radiology, Bayero University, Kano, NG; 4 Department of Radiology, Aminu Kano Teaching Hospital, Kano, NG; 5 Department of Chemical Pathology, Bayero University, Kano, NG; 6 Department of Paediatrics, Bayero University, Kano, NG; 7 Africa Center of Excellence for Population Health and Policy, Bayero University Kano, Kano, NG; 8 Vanderbilt Institute for Global Health (VIGH), Vanderbilt University Medical Center (VUMC), Nashville, Tennessee, USA

**Keywords:** endothelial dysfunction, cardiovascular disease, HIV, microalbuminuria

## Abstract

**Background:**

Endothelial dysfunction constitutes an early pathophysiological event in atherogenesis and cardiovascular disease. This study aimed to assess the prevalence, determinants, and degree of endothelial dysfunction in antiretroviral therapy (ART)–treated people living with HIV (PLWH) in northwestern Nigeria using brachial flow-mediated dilatation (FMD).

**Methods:**

This was a comparative, cross-sectional study. A total of 200 ART-treated adults living with HIV with no evidence of kidney disease were compared with 200 HIV-negative participants attending a tertiary hospital in Kano, Nigeria, between September 2020 and May 2021. Endothelial function was evaluated by measuring FMD with a high-resolution vascular ultrasound transducer. FMD was calculated as the ratio of the brachial artery diameter after reactive hyperemia to baseline diameter and expressed as a percentage of change. Blood and urine samples were obtained from participants in both arms. Urine albumin-to-creatinine ratio (uACR) was calculated using the 2021 CKD-EPI estimated glomerular filtration rate (eGFR) creatinine-cystatin C equation without the race variable, and low-density lipoprotein (LDL) cholesterol was measured using enzymatic method.

**Results:**

The overall mean age (± standard deviation) of the study participants was 42 ± 11 years. Participants in the comparison arm were younger than PLWH (38 ± 11 versus 46 ± 10 years, respectively). The median (interquartile range) uACR was 41.6 (23.2–162.9) mg/g for the ART-treated PLWH versus 14.5 (7.4–27.0) mg/g for healthy controls. PLWH had a significantly lower mean percent FMD when compared to HIV-negative participants (9.8% ± 5.4 versus 12.1% ± 9.2, respectively). Reduced FMD was independently associated with HIV infection (β = –2.83%, 95% CI, –4.44% to –1.21%, *p* = 0.001), estimated glomerular filtration rate (β = –0.04%, 95% CI, –0.07% to –0.01%, *p* = 0.004) and LDL cholesterol (β = –1.12%, 95% CI, –2.13% to –0.11%, *p* = 0.029).

**Conclusion:**

HIV-positive status, lower estimated GFR, and higher LDL cholesterol levels were independently associated with endothelial dysfunction. Future prospective studies with larger cohorts of persons living with HIV (and age- and sex-matched HIV-negative controls) are needed to gain further insight into these important findings. In the interim, aggressive management of modifiable risk factors is warranted.

## Introduction

The HIV pandemic remains a global health problem despite the widespread availability of antiretroviral therapy (ART). There are approximately 37 million people living with HIV (PLWH) globally, out of which approximately 70% reside in sub-Saharan Africa [1,2]. Endothelial dysfunction is the loss of endothelium-dependent vascular relaxation in response to pro-vasodilatory stimuli and constitutes an early pathophysiological event in atherogenesis and cardiovascular diseases (CVD) [3]. PLWH have an increased risk of CVD associated with endothelial dysfunction, and the presence of endothelial dysfunction in them could be an early surrogate risk marker for CVD, especially in persons receiving long-term ART, particularly those receiving ritonavir-boosted protease inhibitors [4,5]. Early diagnosis of endothelial dysfunction in these patients is important to establish preventive interventions, such as lifestyle modifications and pharmacological interventions, where necessary. In PLWH, the occurrence of endothelial dysfunction has been associated with increased expression of adhesion molecules, such as intercellular adhesion molecule-1 (ICAM-1), endothelial adhesion molecule (E-selectin), and inflammatory cytokines, such as tumor necrosis factor-alpha (TNF-α) and interleukin (IL-6) [6].

The detection of abnormalities in clinical and laboratory parameters related to HIV and associated endothelial dysfunction could also provide clues regarding the etiology of these early stages of atherosclerosis [7]. Modern risk assessment tools frequently underestimate CVD risk profiles and have not included substantial numbers of ART-treated PLWH [8] Various cardiovascular risk assessment tools are available, including the Framingham risk score, Systematic Coronary Risk Evaluation (SCORE), the ACC/AHA ASCVD pooled cohort equations (PCE), the 2018 Cholesterol Clinical Practice Guidelines, the World Health Organization (WHO) risk prediction charts, and the 2017 Hypertension Clinical Practice Guidelines. However, it is important to note that these tools often underestimate CVD risk due to their validation for specific populations. For example, the PCE is widely validated and applicable to the general US clinical population. Nevertheless, it may systematically underestimate the risk for individuals from certain racial/ethnic groups outside the United States. This discrepancy arises from population heterogeneity, variations in risk factor prevalence, and differing hazards related to atherosclerotic cardiovascular disease. Furthermore, these tools may also underestimate risk for persons with lower socioeconomic status or individuals with chronic inflammatory diseases like HIV, rheumatoid arthritis, and sarcoidosis. Therefore, it is important to recalibrate and not interchangeably use these cardiovascular risk assessment tools, given the distinct risk factor profiles such as hypertension burden, levels of total cholesterol, prevalence of metabolic disorders, and diverse CVD profiles between Western and other populations.

Although endothelial function has been extensively studied by others, there is a paucity of data on endothelial function among ART-treated PLWH. Therefore, the aim of this study was to assess the prevalence, determinants, and degree of endothelial dysfunction in adult Nigerian PLWH by using brachial flow-mediated dilatation (FMD).

## Methods

This is a cross-sectional study involving 400 participants recruited from the Aminu Kano Teaching Hospital in Kano, Nigeria. A total of 200 adults (≥18 years of age) diagnosed with HIV and receiving ART with no evidence of kidney disease (i.e., having an eGFR > 60 ml/min/1.73m^2^) were compared with 200 healthy sex- and age-matched HIV-negative participants receiving longitudinal care between September 2020 and May 2021. We excluded persons with a history of an active opportunistic infection (i.e., pulmonary tuberculosis, candida esophagitis, cryptococcal meningitis, *Pneumocystis jiroveci* pneumonia, etc.) within the past three months, pregnant women, and persons with a known history of hypertension, diabetes mellitus, malignancy, and/or heart disease.

We employed a structured questionnaire and trained research assistants to gather data on sociodemographics, comorbidities, viro-immunological profiles, coinfections, and HIV-related clinical manifestations, as well as current and previous antiretroviral medications. To supplement this information, we also reviewed relevant medical and laboratory records stored within the HIV clinic.

Urine albumin-creatinine ratio (uACR) was determined from two consecutive early-morning urine samples collected in the clinic three months apart. Urine albumin was measured by immunonephelometry using the BN II system (Siemens, Healthcare Diagnostics, Marburg, Germany) and urine creatinine by the Jaffe kinetic reaction with picric acid (ADVIA Chemistry, Siemens, Healthcare Diagnostics, NY, USA).

Albuminuria was defined as uACR of more than 30 mg/g, as assessed from random urine collection. Microalbuminuria was defined as uACR between 30 and 300 mg/g and macroalbuminuria as uACR of more than 300 mg/g [9]. Blood samples were collected after an eight-hour overnight fast, processed by centrifugation, and stored at –80°C. These samples were subsequently batch analyzed for glucose, total cholesterol, HDL cholesterol, direct LDL cholesterol, triglycerides, creatinine, CD4 cell count, HIV plasma viral load, and serum cystatin C using enzymatic colorimetric methods on Abbott Architect c4000autoanalyzer (Abbott in vitro diagnostics, Illinois, USA). Analytic quality was assured by analyzing quality control sample levels 1, 2, and 3 with each assay run. Batch samples whose control values fall outside the established laboratory values (>±2 SD) were rejected.

In preparation for the carotid Doppler ultrasonography and brachial artery flow-mediated dilation (FMD) measurements, the study participants abstained from alcohol, caffeine, and/or nicotine 12 hours prior to the examination and only consumed clear liquids on the morning of the examination. The scan was performed with a 7.5 MHz linear transducer (Nemio XG, Toshiba Medical Systems SA), in a supine position.

We determined the degree of endothelial dysfunction by measuring AFMD. As described by Corretti et al. [10], the initial baseline blood pressure of the patient was first measured using a digital blood pressure monitor. With gentle right arm abduction, the ultrasound gel was applied on the medial aspect of the arm at about 5 cm above the antecubital fossa. The brachial artery was imaged at this level. The brachial artery diameter was obtained by taking measurements from the intima-lumen interface of the near wall to the intima-lumen interface of the far wall. Hyperemia was then provoked by inflating the sphygmomanometer cuff placed on the patient’s right forearm up to 50 mmHg above systolic pressure for five minutes and then deflating it. The post-occlusive diameter was obtained by repeating the same measurement at 60 seconds. Three measurements were taken over the 1 cm length of the brachial artery and their average obtained. The AFMD was then calculated as a ratio of the difference between two diameters with the baseline diameter. The result was then expressed as a percentage. The median (interquartile range [IQR]) intra-observer intersession percentage of variation for brachial artery diameter was 2.38% (0–5.71), similar to previous studies [7,11].

As described by Allan [12], the carotid intima-media thickness was assessed in a supine position with a pillow under the shoulders of the patients. The intima-media thickness was measured at the upper third of the common carotid artery at the posterior wall, 1–2 cm below the carotid bifurcation. A minimum of three measurements were taken, and their average was recorded. The intima-media thickness’s normal value was less than 0.8 mm.

The study was approved by the Ethics Committee of Aminu Kano Teaching Hospital (EC-2840). Informed consent was obtained from all participants prior to enrollment.

### Statistical analysis

Continuous variables were presented as means ± SD or as medians and interquartile ranges (IQRs), as appropriate, while categorical data were reported as proportions. An independent *t*-test or Wilcoxon rank-sum test compared continuous variables between PLWH and participants without HIV, whereas Pearson’s or Fisher exact tests were used for comparing proportions. Multivariable linear regression models were used to determine the effect of independent predictors on flow-mediated dilation (FMD) of the brachial artery and log-10 transformed common carotid intima-media thickness. Variables were added to the multivariable linear regression model by using a stepwise regression strategy; a backward elimination method was used to include variables with prespecified *p*-values of less than 0.20 into the model. Variables that were biologically plausible to be associated with FMD of the brachial artery and log-10 transformed common carotid intima-media thickness (cIMT) were added to the model, even if their *p*-values were >0.20. A *p*-value of <0.05 was considered statistically significant at 95% confidence interval. All analyses were performed using STATA, version 12 (STATA Corp., College Station, TX, USA).

## Results

A total of 200 PLWH and 200 HIV-negative age- and sex-matched controls were evaluated. The overall mean age of the study participants was 42 ± 11 years. Participants in the control arm were younger than PLWH, mean age ± SD: 38 ± 11 versus 46 ± 10 years, respectively (Table 1). The median uACR for HIV-positive participants was 41.6 (23.2–162.9) versus 14.5 (7.4–27.0) for those in the control arm. The mean serum cystatin C level was significantly higher in PLWH than in healthy controls (1.00 ± 0.43 versus 0.79 ± 0.23, *P* < 0.0001). Approximately 97% of PLWH in the study were virally suppressed.

**Table 1:** Baseline characteristics of participants by HIV status, Kano, Nigeria.


COVARIATE	ALL PARTICIPANTS (*n* =400)	HIV POSITIVE (*n* = 200)	HIV NEGATIVE (*n* = 200)	*p*-VALUE

Age (years)	42 ± 11	46 ± 10	38 ± 11	< 0.0001

Sex *n* (%)				

Male	210 (52.5)	102 (51.0)	108 (54.0)	

Female	190 (47.5)	98 (49.0)	92 (46.0)	0.62

BMI	24.0 ± 4.6	23.9 ± 5.3	24.0 ± 3.8	0.80

Systolic BP (mm Hg)	114 ± 18	114 ± 22	112 ± 12	0.16

Diastolic BP (mm Hg)	75 ± 12	77 ± 15	74 ± 9	0.049

Total cholesterol (mmol/L)	4.85 ± 1.02	4.85 ± 1.18	4.85 ± 0.84	0.98

HDL-C (mmol/L)	0.88 (0.58–1.23)	0.73 (0.44–1.27)	0.94 (0.7–1.21)	0.003

LDL-C (mmol/L)	3.31 ± 0.94	3.32 ± 1.04	3.29 ± 0.82	0.69

Total cholesterol categories				

Desirable (<5.2 mmol/L)	262 (65.5)	125 (62.5)	137 (68.5)	0.08

Borderline high (5.2–6.1 mmol/L)	94 (65.5)	46 (23.0)	48 (24.0)	

High(>6.1 mmol/L)	44 (23.5)	29 (14.5)	15 (7.5)	

Optimal LDL (<2.6 mmol/L)	81 (20.2)	46 (23.0)	35 (17.5)	0.17

Best HDL	42 (10.5)	23 (11.5)	19 (9.5)	0.52

Ever smoked, *n* (%)	10 (2.5)	10 (5.5)	0 (0.0)	

HTN *n* (%)	43 (10.7)	43 (21.5)	0 (0.0)	

Diabetes mellitus *n* (%)	10 (2.5)	10 (5.0)	0 (0.0)	

Cystatin C	0.90 ± 0.36	1.00 ± 0.43	0.79 ± 0.23	< 0.0001

uACR	25.9 (10.5–54.9)	41.6 (23.2–162.9)	14.5 (7.4–27.0)	< 0.0001

ACR categories, *n* (%)				

Normoalbuminuria	225 (56.3)	71 (35.5)	154 (77.0)	< 0.001

Microalbuminuria	136 (34.0)	90 (45.0)	46 (23.0)	

Macroalbuminuria	39 (9.8)	39 (19.5)	0 (0.0)	

Albuminuria (uACR ≥ 30 mg/g), *n* (%)	175 (43.8)	129 (64.5)	46 (23.0)	< 0.001

eGFR (ml/min/1.73m^2^)	110.9 ± 30.6	99.2 ± 29.1	122.6 ± 27.5	< 0.0001

eGFR (<60ml/min), *n* (%)	21 (5.3)	19 (9.5)	2 (1.0)	< 0.001

cIMT = carotid artery intima media thickness	0.71 ± 0.34	0.74 ± 0.45	0.68 ± 0.15	0.08

AFMD	10.96 ± 7.65	9.82 ± 5.43	12.09 ± 9.23	0.003

HIV RNA, log copies/mL	–	3.31 ± 1.10	–	

HIV-1 RNA (viral load) suppressed, *n* (%)	–	193 (96.5)	–	–

CD4+ cell count, cells/mm^3^	–	433 (298–592)	–	–


uACR = urine albumin-to-creatinine ratio, FMD = brachial artery flow-mediated dilation, cIMT = carotid artery intima media thickness, HDL = high-density lipoprotein cholesterol, LDL = low-density lipoprotein cholesterol, eGFR = estimated glomerular filtration rate.

The overall prevalence of albuminuria (uACR > 30) was 43.8%. The proportion of participants with microalbuminuria was higher in PLWH than in healthy controls (45.0% versus 23.0%, respectively; *p* < 0.001). The prevalence of macroalbuminuria among PLWH was 19.5%. No case of macroalbuminuria was reported among the controls. More than half of the study participants (65.5%) had desirable total cholesterol (<5.2 mmol/L).

PLWH had significantly lower mean percent FMD than non-HIV participants (9.82% ± 5.43% versus 12.09% ± 9.23%, respectively). In unadjusted univariable analysis, HIV infection was significantly associated with lower FMD (β = –2.28%, 95% CI, –3.77% to –0.79%, *p* = 0.003), and this association persisted after adjusting for potential cofounding variables (β = –2.83%, 95% CI, –4.44% to –1.21%, *p* = 0.001). Whereas estimated GFR (β = –0.02%, 95% CI, –0.04% to 0.01%, *p* = 0.13 and LDL (β = –0.32%, 95% CI, –1.12% to 0.48%, *p* = 0.43) were not significantly associated with lower FMD with univariate analysis, this association became accentuated and significant on multivariable analysis (β = –0.04%, 95% CI, –0.07% to –0.01%, *p* = 0.004) and (β = –1.12%, 95% CI, –2.13% to –0.11%, *p* = 0.029) (Table 2).

**Table 2:** Factors associated with brachial artery flow-mediated dilation, Kano, Nigeria.


VARIABLE	UNADJUSTED MODEL				ADJUSTED MODEL			
	
	β-COEFFICIENT	SE	95% CI	*p*-VALUE	β-COEFFICIENT	SE	95% CI	*p*-VALUE

HIV positive	–2.28	0.76	–3.77, –0.79	0.003	–2.83	0.82	–4.44, –1.21	0.001

Age (years)	–0.03	0.03	–0.09, 0.04	0.45	–0.04	0.04	–0.121, 0.03	0.27

Diastolic BP (mm Hg)	–0.02	0.03	–0.08, 0.04	0.48	–0.05	0.03	–0.11, 0.02	0.14

eGFR (ml/min/1.73 m^2^)	–0.02	0.01	–0.04, 0.01	0.13	–0.04	0.02	–0.07, –0.01	0.004

Total cholesterol (mmol/L)	0.71	0.37	–0.02, 1.44	0.057	1.34	0.48	0.41, 2.28	0.005

LDL cholesterol (mmol/L)	–0.32	0.41	–1.12, 0.48	0.43	–1.12	0.51	–2.13, –0.11	0.029


BP = blood pressure, CI = confidence interval, LDL = low-density lipoprotein, SE = standard error, eGFR = estimated glomerular filtration.

No significant association was found between HIV infection and common coronary artery intima-media thickness (β = 0.003, 95% CI, –0.056 to 0.050, *p* = 0.90. With univariate analysis, higher log-10 transformed cIMT was associated with increasing age (β = 0.008, 95% CI, 0.006 to 0.010, *p* < 0.001) and systolic blood pressure (β = 0.003, 95% CI, 0.001 to 0.004, *p* < 0.001). After adjusting for potential confounders, higher log-10 cIMT remained independently associated with increasing age (β = 0.008, 95% CI, 0.006 to 0.010, *p* < 0.001) and systolic blood pressure (β = 0.002, 95% CI, 0.0002 to 0.0029, *p* = 0.024) (Table 3).

**Table 3:** Factors associated with common carotid intima-media thickness, Kano, Nigeria.


VARIABLE	UNADJUSTED MODEL				ADJUSTED MODEL			
	
	β-COEFFICIENT	SE	95% CI	*p*-VALUE	β-COEFFICIENT	SE	95% CI	*p*-VALUE

HIV positive	0.046	0.025	–0.002, 0.095	0.059	–0.003	0.027	–0.056, 0.050	0.901

Age (years)	0.008	0.001	0.0058, 0.0099	< 0.001	0.008	0.001	0.0055, 0.010	< 0.001

Systolic BP (mm Hg)	0.003	0.001	0.0011, 0.0038	< 0.001	0.002	0.001	0.0002, 0.0029	0.024

Total cholesterol (mmol/L)	0.002	0.012	–0.022, 0.0255	0.90	–0.013	0.015	–0.0429, 0.0153	0.351

LDL cholesterol (mmol/L)	–0.006	0.013	–0.0320, 0.0199	0.65	–0.005	0.016	–0.0356, 0.0153	0.757

Albuminuria (uACR > 30 mg/g)	0.015	0.025	–0.0344, 0.0637	0.56	–0.011	0.026	–0.061, 0.0396	0.666

Diabetes mellitus	0.019	0.079	–0.1367, 0.1751	0.81	0.102	0.075	–0.0459, 0.250	0.176


Dependent variable is log-10 cIMT, BP = blood pressure, LDL = low-density lipoprotein, SE = standard error, uACR=urine albumin-to-creatinine ratio.

## Discussion

Endothelial dysfunction is an important factor in the initiation and clinical manifestation of atherosclerosis and a known early predictor of future cardiovascular events in patients with or without known cardiovascular disease risk factors [13]. Noninvasive endothelial function assessments continue to gain attention in preventive cardiovascular medicine among PLWH [14]. In this cross-sectional study, we found that 10% of ART-treated PLWH had noninvasive evidence of endothelial dysfunction, consistent with previous studies [13,15,16]. However, some studies have not been able to establish an association between HIV status and endothelial dysfunction among ART-experienced populations [13,17].

The natural history of endothelial function PLWH remains poorly understood, and whether endothelial dysfunction is a consequence of HIV infection, exposure to long-term ART, or both remains to be elucidated [18]. HIV infection is associated with increased systemic inflammation and a hypercoagulable state, both of which may be secondary to increased monocytes migrating across the endothelium and forming foam cells, thereby contributing to endothelial dysfunction. Additional studies are warranted to better understand the pathogenesis of endothelial dysfunction among ART-treated PLWH.

Urine albumin-to-creatinine ratio (uACR) is an important measure of kidney function and an independent risk factor for cardiovascular disease [19]. We observed a twofold difference in proportions of persons with microalbuminuria between ART-treated PLWH (45.0%) compared to HIV-negative controls (23.0%), translating to an association between HIV infection and albuminuria. Several hypotheses have been proposed to explain mechanisms that can lead to albuminuria in PLWH. Specifically, albuminuria has been hypothesized to be due to HIV directly damaging the glomeruli, causing HIV nephropathy (HIVAN); deposition of immune complexes generated as an immune response to the HIV; opportunistic infections that lead to glomerular damage [20]; and/or the side effects of ART that may directly affect the kidneys, e.g., tenofovir disoproxil fumarate (tubular insult) [21], or cause higher rates of hyperlipidaemia and an increased tendency toward atherosclerotic changes [22].

The relationship between HIV infection and its treatment and cardiovascular risk is well established; however, we failed to find an association between HIV and cIMT. Our finding is in agreement with Fourie et al. [23], who also failed to detect subclinical atherosclerosis in their HIV-positive participants, but in contrast to several studies that indicate higher cIMT levels among PLWH [24,25]. The difference in findings may be explained by the fact that we measured cIMT in the common carotid artery, while the other studies performed their assessments mainly in the carotid bifurcation region [23].

We found an association between HIV-positive status and endothelial dysfunction. FMD was significantly reduced in seropositive individuals compared to controls, consistent with findings by others [26,27,28,29]. However, our report is at variance with other studies that failed to establish an association between HIV infection and endothelial dysfunction [13,17]. The underlying reasons to explain this variance are not known. We speculate that types of ART regimens and ethnic variations may play a role. Meanwhile, larger prospective studies are needed to confirm or refute our findings.

Both participant-related and environmental factors could lead to low reproducibility of FMD measurements [30]. Therefore, in this study, we ensured reproducible measurements and a low coefficient of variation for FMD by adopting standardized methodologies for measuring FMD, as described [17,31]. Nevertheless, this study may have been limited by the fact that we were unable to account for the use of certain medications that could affect FMD, such as anticoagulants or HMG-CoA reductase inhibitors (statins). As a pooled cohort study, we were unable to assess the effects of individual ART classes due to the wide range of regimen options in the cohort and limited sample sizes of specific ART regimens. We also could not discount the potential effects of unmeasured residual confounders or underestimate the effects of CVD risk factors on FMD due to its variability as a biological measure, even when measured using highly standardized protocols. Also, measurement variation in ultrasound machines and sonographers may have limited our ability to discern the effects of individual markers of HIV disease severity on FMD. Finally, we used a wide range of ages (five-year band) to match HIV-positive participants and controls, which may not have been optimal.

## Conclusion

We found impaired endothelial function to be independently associated with HIV-positive status, estimated glomerular filtration rate, and LDL cholesterol levels. Future larger prospective studies are needed to further elucidate the pathoetiological mechanisms underlying these findings.
